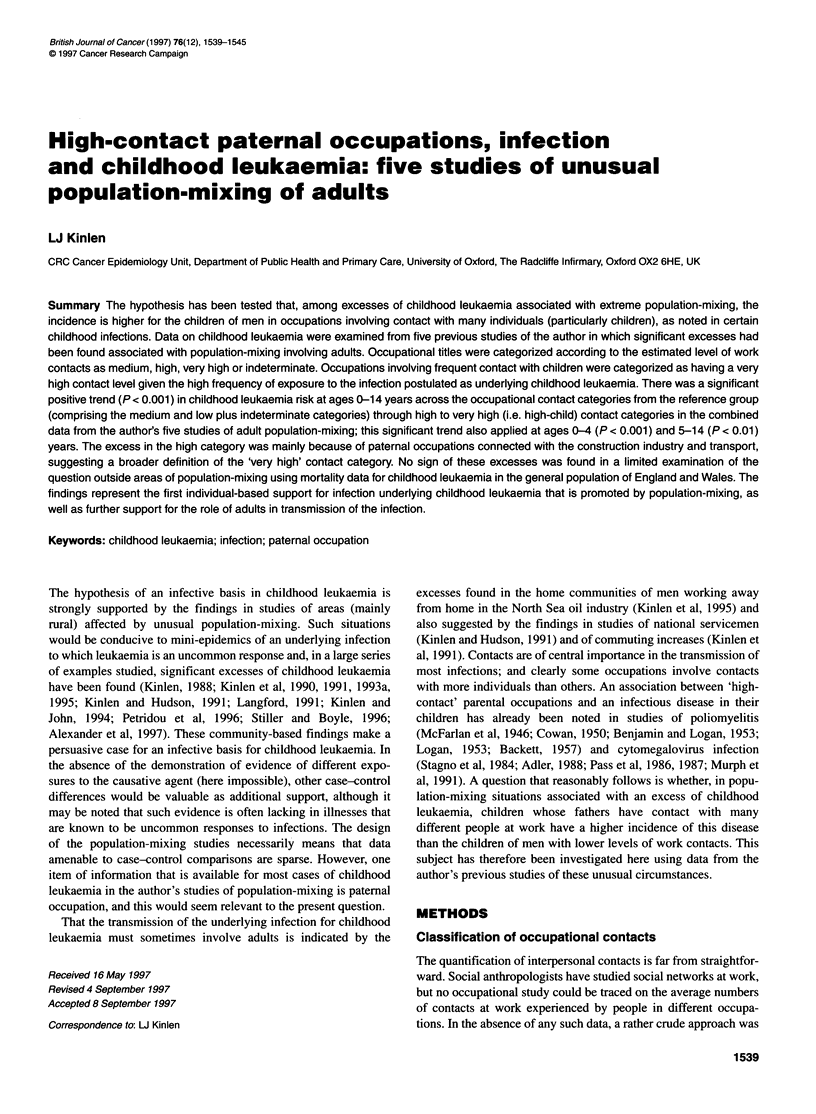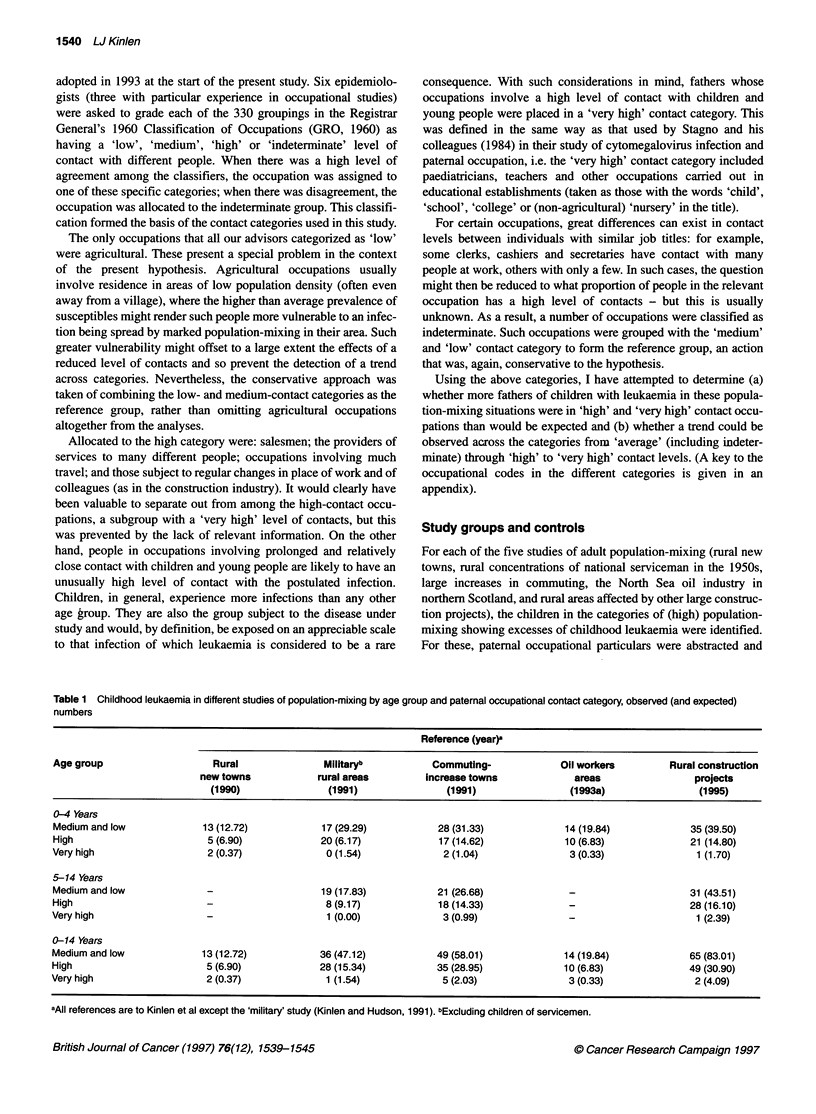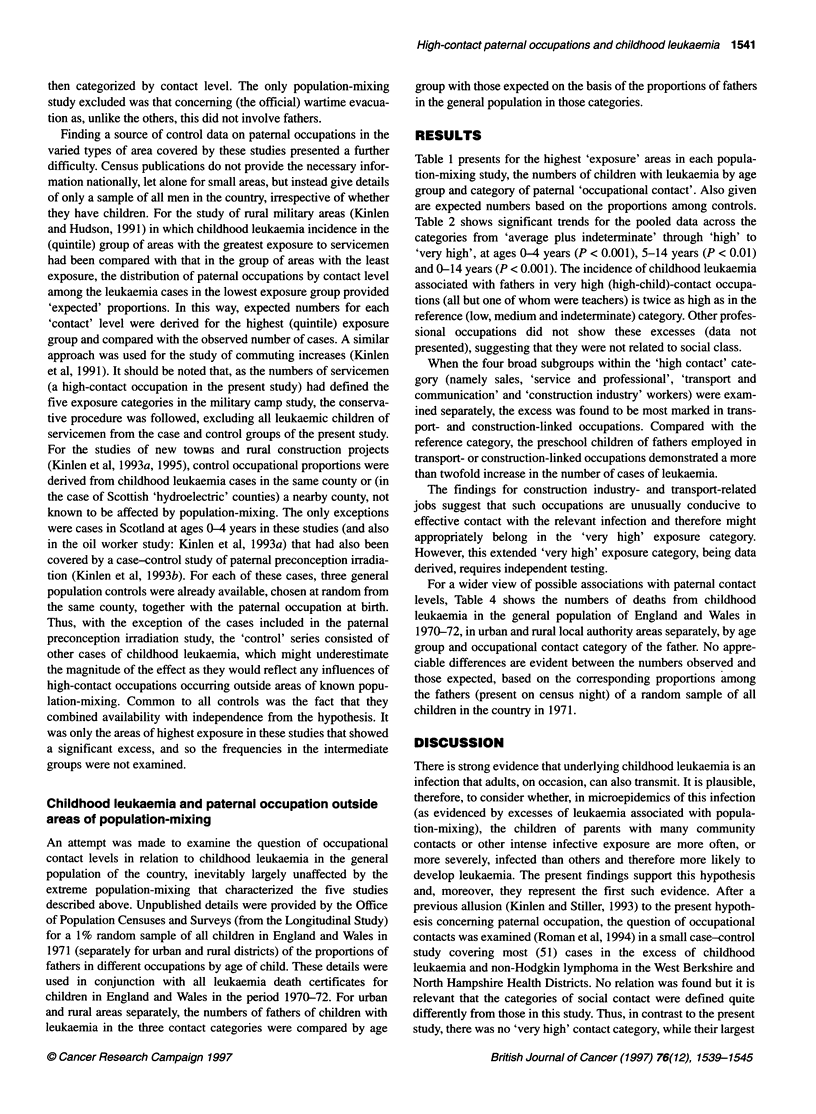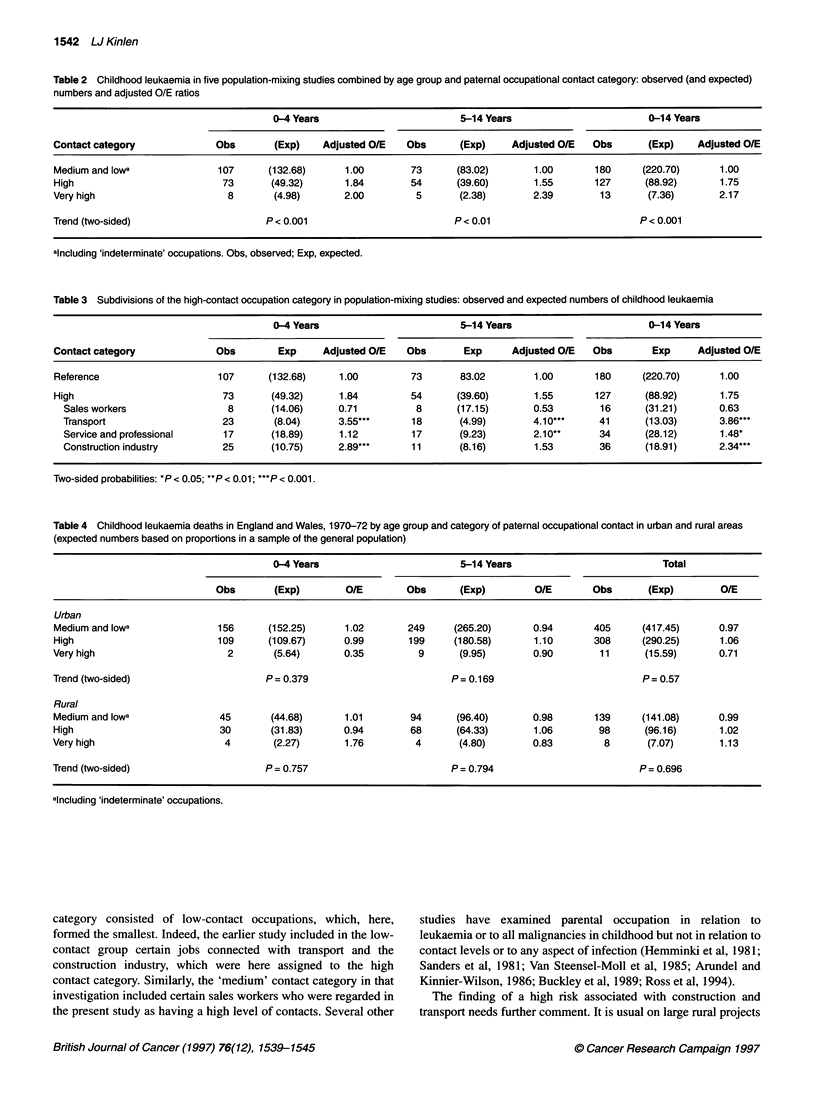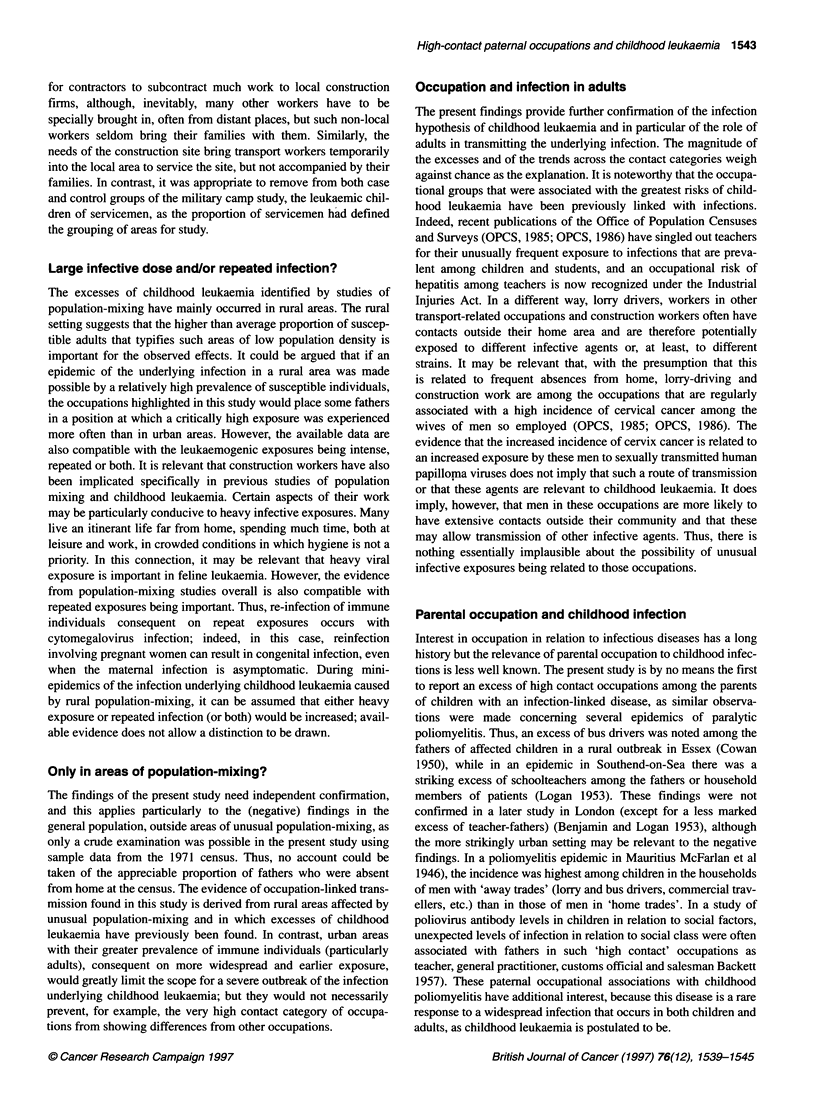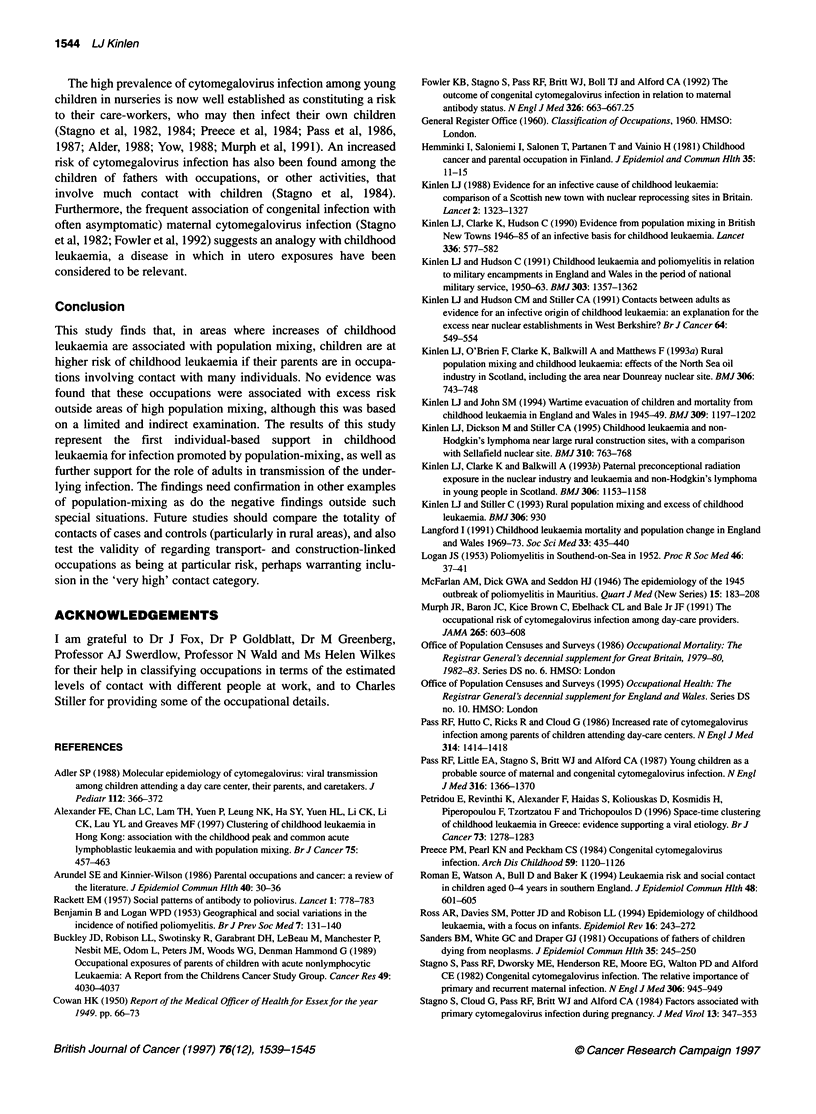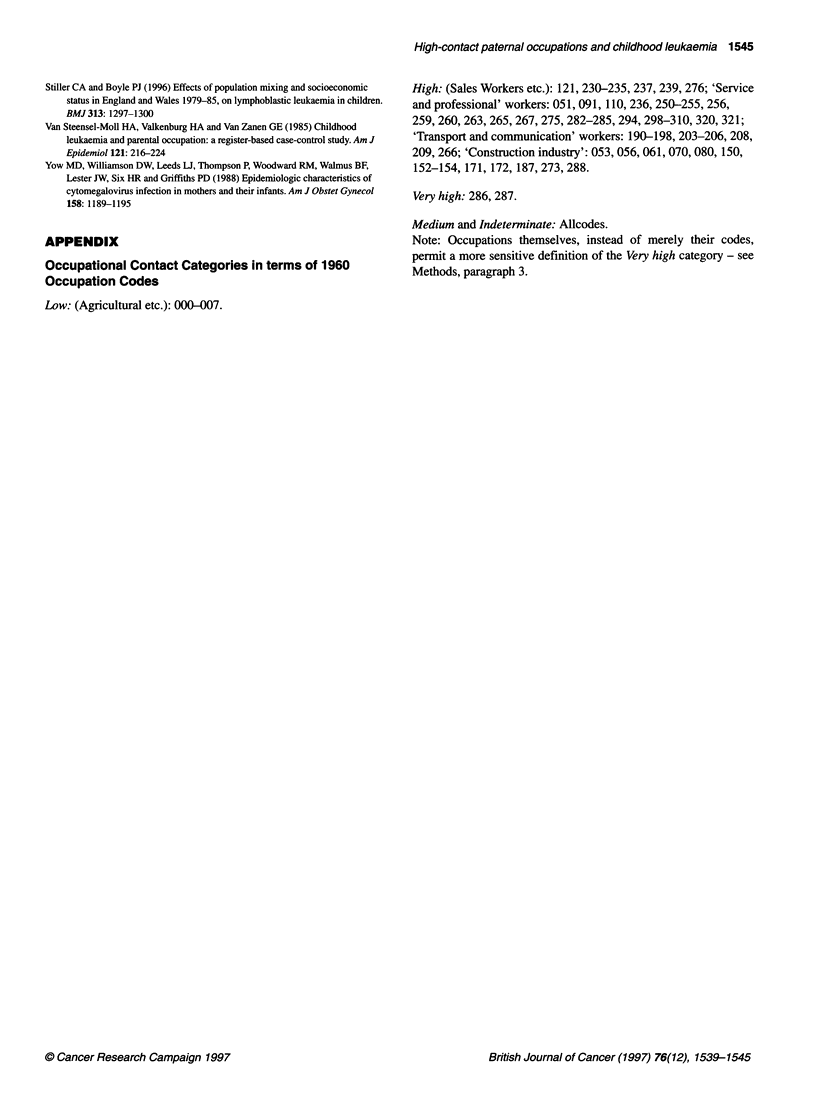# High-contact paternal occupations, infection and childhood leukaemia: five studies of unusual population-mixing of adults.

**DOI:** 10.1038/bjc.1997.592

**Published:** 1997

**Authors:** L. J. Kinlen

**Affiliations:** Department of Public Health and Primary Care, University of Oxford, The Radcliffe Infirmary, UK.

## Abstract

The hypothesis has been tested that, among excesses of childhood leukaemia associated with extreme population-mixing, the incidence is higher for the children of men in occupations involving contact with many individuals (particularly children), as noted in certain childhood infections. Data on childhood leukaemia were examined from five previous studies of the author in which significant excesses had been found associated with population-mixing involving adults. Occupational titles were categorized according to the estimated level of work contacts as medium, high, very high or indeterminate. Occupations involving frequent contact with children were categorized as having a very high contact level given the high frequency of exposure to the infection postulated as underlying childhood leukaemia. There was a significant positive trend (P < 0.001) in childhood leukaemia risk at ages 0-14 years across the occupational contact categories from the reference group (comprising the medium and low plus indeterminate categories) through high to very high (i.e. high-child) contact categories in the combined data from the author's five studies of adult population-mixing; this significant trend also applied at ages 0-4 (P < 0.001) and 5-14 (P < 0.01) years. The excess in the high category was mainly because of paternal occupations connected with the construction industry and transport, suggesting a broader definition of the 'very high' contact category. No sign of these excesses was found in a limited examination of the question outside areas of population-mixing using mortality data for childhood leukaemia in the general population of England and Wales. The findings represent the first individual-based support for infection underlying childhood leukaemia that is promoted by population-mixing, as well as further support for the role of adults in transmission of the infection.


British Journal of Cancer (1997) 76(12), 1539–1545
© 1997 Cancer Research Campaign

### High-contact paternal occupations, infection and childhood leukaemia: five studies of unusual population-mixing of adults

**LJ Kinlen**

CRC Cancer Epidemiology Unit, Department of Public Health and Primary Care, University of Oxford, The Radcliffe Infirmary, Oxford OX2 6HE, UK

**Summary** The hypothesis has been tested that, among excesses of childhood leukaemia associated with extreme population-mixing, the incidence is higher for the children of men in occupations involving contact with many individuals (particularly children), as noted in certain childhood infections. Data on childhood leukaemia were examined from five previous studies of the author in which significant excesses had been found associated with population-mixing involving adults. Occupational titles were categorized according to the estimated level of work contacts as medium, high, very high or indeterminate. Occupations involving frequent contact with children were categorized as having a very high contact level given the high frequency of exposure to the infection postulated as underlying childhood leukaemia. There was a significant positive trend ($P < 0.001$) in childhood leukaemia risk at ages 0–14 years across the occupational contact categories from the reference group (comprising the medium and low plus indeterminate categories) through high to very high (i.e. high-child) contact categories in the combined data from the author's five studies of adult population-mixing; this significant trend also applied at ages 0–4 ($P < 0.001$) and 5–14 ($P < 0.01$) years. The excess in the high category was mainly because of paternal occupations connected with the construction industry and transport, suggesting a broader definition of the 'very high' contact category. No sign of these excesses was found in a limited examination of the question outside areas of population-mixing using mortality data for childhood leukaemia in the general population of England and Wales. The findings represent the first individual-based support for infection underlying childhood leukaemia that is promoted by population-mixing, as well as further support for the role of adults in transmission of the infection.

**Keywords:** childhood leukaemia; infection; paternal occupation

The hypothesis of an infective basis in childhood leukaemia is strongly supported by the findings in studies of areas (mainly rural) affected by unusual population-mixing. Such situations would be conducive to mini-epidemics of an underlying infection to which leukaemia is an uncommon response and, in a large series of examples studied, significant excesses of childhood leukaemia have been found (Kinlen, 1988; Kinlen et al, 1990, 1991, 1993a, 1995; Kinlen and Hudson, 1991; Langford, 1991; Kinlen and John, 1994; Petridou et al, 1996; Stiller and Boyle, 1996; Alexander et al, 1997). These community-based findings make a persuasive case for an infective basis for childhood leukaemia. In the absence of the demonstration of evidence of different exposures to the causative agent (here impossible), other case–control differences would be valuable as additional support, although it may be noted that such evidence is often lacking in illnesses that are known to be uncommon responses to infections. The design of the population-mixing studies necessarily means that data amenable to case–control comparisons are sparse. However, one item of information that is available for most cases of childhood leukaemia in the author's studies of population-mixing is paternal occupation, and this would seem relevant to the present question.

That the transmission of the underlying infection for childhood leukaemia must sometimes involve adults is indicated by the excesses found in the home communities of men working away from home in the North Sea oil industry (Kinlen et al, 1995) and also suggested by the findings in studies of national servicemen (Kinlen and Hudson, 1991) and of commuting increases (Kinlen et al, 1991). Contacts are of central importance in the transmission of most infections; and clearly some occupations involve contacts with more individuals than others. An association between 'high-contact' parental occupations and an infectious disease in their children has already been noted in studies of poliomyelitis (McFarlan et al, 1946; Cowan, 1950; Benjamin and Logan, 1953; Logan, 1953; Backett, 1957) and cytomegalovirus infection (Stagno et al, 1984; Adler, 1988; Pass et al, 1986, 1987; Murph et al, 1991). A question that reasonably follows is whether, in population-mixing situations associated with an excess of childhood leukaemia, children whose fathers have contact with many different people at work have a higher incidence of this disease than the children of men with lower levels of work contacts. This subject has therefore been investigated here using data from the author's previous studies of these unusual circumstances.

*Received 16 May 1997*
*Revised 4 September 1997*
*Accepted 8 September 1997*

*Correspondence to:* LJ Kinlen

### METHODS

### Classification of occupational contacts

The quantification of interpersonal contacts is far from straightforward. Social anthropologists have studied social networks at work, but no occupational study could be traced on the average numbers of contacts at work experienced by people in different occupations. In the absence of any such data, a rather crude approach was adopted in 1993 at the start of the present study. Six epidemiologists (three with particular experience in occupational studies) were asked to grade each of the 330 groupings in the Registrar General's 1960 Classification of Occupations (GRO, 1960) as having a 'low', 'medium', 'high' or 'indeterminate' level of contact with different people. When there was a high level of agreement among the classifiers, the occupation was assigned to one of these specific categories; when there was disagreement, the occupation was allocated to the indeterminate group. This classification formed the basis of the contact categories used in this study.

The only occupations that all our advisors categorized as 'low' were agricultural. These present a special problem in the context of the present hypothesis. Agricultural occupations usually involve residence in areas of low population density (often even away from a village), where the higher than average prevalence of susceptibles might render such people more vulnerable to an infection being spread by marked population-mixing in their area. Such greater vulnerability might offset to a large extent the effects of a reduced level of contacts and so prevent the detection of a trend across categories. Nevertheless, the conservative approach was taken of combining the low- and medium-contact categories as the reference group, rather than omitting agricultural occupations altogether from the analyses.

Allocated to the high category were: salesmen; the providers of services to many different people; occupations involving much travel; and those subject to regular changes in place of work and of colleagues (as in the construction industry). It would clearly have been valuable to separate out from among the high-contact occupations, a subgroup with a 'very high' level of contacts, but this was prevented by the lack of relevant information. On the other hand, people in occupations involving prolonged and relatively close contact with children and young people are likely to have an unusually high level of contact with the postulated infection. Children, in general, experience more infections than any other age group. They are also the group subject to the disease under study and would, by definition, be exposed on an appreciable scale to that infection of which leukaemia is considered to be a rare consequence. With such considerations in mind, fathers whose occupations involve a high level of contact with children and young people were placed in a 'very high' contact category. This was defined in the same way as that used by Stagno and his colleagues (1984) in their study of cytomegalovirus infection and paternal occupation, i.e. the 'very high' contact category included paediatricians, teachers and other occupations carried out in educational establishments (taken as those with the words 'child', 'school', 'college' or (non-agricultural) 'nursery' in the title).

For certain occupations, great differences can exist in contact levels between individuals with similar job titles: for example, some clerks, cashiers and secretaries have contact with many people at work, others with only a few. In such cases, the question might then be reduced to what proportion of people in the relevant occupation has a high level of contacts – but this is usually unknown. As a result, a number of occupations were classified as indeterminate. Such occupations were grouped with the 'medium' and 'low' contact category to form the reference group, an action that was, again, conservative to the hypothesis.

Using the above categories, I have attempted to determine (a) whether more fathers of children with leukaemia in these population-mixing situations were in 'high' and 'very high' contact occupations than would be expected and (b) whether a trend could be observed across the categories from 'average' (including indeterminate) through 'high' to 'very high' contact levels. (A key to the occupational codes in the different categories is given in an appendix).

### Study groups and controls

For each of the five studies of adult population-mixing (rural new towns, rural concentrations of national serviceman in the 1950s, large increases in commuting, the North Sea oil industry in northern Scotland, and rural areas affected by other large construction projects), the children in the categories of (high) population-mixing showing excesses of childhood leukaemia were identified. For these, paternal occupational particulars were abstracted and

**Table 1** Childhood leukaemia in different studies of population-mixing by age group and paternal occupational contact category, observed (and expected) numbers

| Age group | Reference (year)[a] | | | | |
|---|---|---|---|---|---|
| | Rural new towns (1990) | Military[b] rural areas (1991) | Commuting-increase towns (1991) | Oil workers areas (1993a) | Rural construction projects (1995) |
| *0–4 Years* | | | | | |
| Medium and low | 13 (12.72) | 17 (29.29) | 28 (31.33) | 14 (19.84) | 35 (39.50) |
| High | 5 (6.90) | 20 (6.17) | 17 (14.62) | 10 (6.83) | 21 (14.80) |
| Very high | 2 (0.37) | 0 (1.54) | 2 (1.04) | 3 (0.33) | 1 (1.70) |
| *5–14 Years* | | | | | |
| Medium and low | – | 19 (17.83) | 21 (26.68) | – | 31 (43.51) |
| High | – | 8 (9.17) | 18 (14.33) | – | 28 (16.10) |
| Very high | – | 1 (0.00) | 3 (0.99) | – | 1 (2.39) |
| *0–14 Years* | | | | | |
| Medium and low | 13 (12.72) | 36 (47.12) | 49 (58.01) | 14 (19.84) | 65 (83.01) |
| High | 5 (6.90) | 28 (15.34) | 35 (28.95) | 10 (6.83) | 49 (30.90) |
| Very high | 2 (0.37) | 1 (1.54) | 5 (2.03) | 3 (0.33) | 2 (4.09) |

[a]All references are to Kinlen et al except the 'military' study (Kinlen and Hudson, 1991). [b]Excluding children of servicemen.

© Cancer Research Campaign 1997

then categorized by contact level. The only population-mixing study excluded was that concerning (the official) wartime evacuation as, unlike the others, this did not involve fathers.

Finding a source of control data on paternal occupations in the varied types of area covered by these studies presented a further difficulty. Census publications do not provide the necessary information nationally, let alone for small areas, but instead give details of only a sample of all men in the country, irrespective of whether they have children. For the study of rural military areas (Kinlen and Hudson, 1991) in which childhood leukaemia incidence in the (quintile) group of areas with the greatest exposure to servicemen had been compared with that in the group of areas with the least exposure, the distribution of paternal occupations by contact level among the leukaemia cases in the lowest exposure group provided 'expected' proportions. In this way, expected numbers for each 'contact' level were derived for the highest (quintile) exposure group and compared with the observed number of cases. A similar approach was used for the study of commuting increases (Kinlen et al, 1991). It should be noted that, as the numbers of servicemen (a high-contact occupation in the present study) had defined the five exposure categories in the military camp study, the conservative procedure was followed, excluding all leukaemic children of servicemen from the case and control groups of the present study. For the studies of new towns and rural construction projects (Kinlen et al, 1993*a*, 1995), control occupational proportions were derived from childhood leukaemia cases in the same county or (in the case of Scottish 'hydroelectric' counties) a nearby county, not known to be affected by population-mixing. The only exceptions were cases in Scotland at ages 0–4 years in these studies (and also in the oil worker study: Kinlen et al, 1993*a*) that had also been covered by a case–control study of paternal preconception irradiation (Kinlen et al, 1993*b*). For each of these cases, three general population controls were already available, chosen at random from the same county, together with the paternal occupation at birth. Thus, with the exception of the cases included in the paternal preconception irradiation study, the 'control' series consisted of other cases of childhood leukaemia, which might underestimate the magnitude of the effect as they would reflect any influences of high-contact occupations occurring outside areas of known population-mixing. Common to all controls was the fact that they combined availability with independence from the hypothesis. It was only the areas of highest exposure in these studies that showed a significant excess, and so the frequencies in the intermediate groups were not examined.

### Childhood leukaemia and paternal occupation outside areas of population-mixing

An attempt was made to examine the question of occupational contact levels in relation to childhood leukaemia in the general population of the country, inevitably largely unaffected by the extreme population-mixing that characterized the five studies described above. Unpublished details were provided by the Office of Population Censuses and Surveys (from the Longitudinal Study) for a 1% random sample of all children in England and Wales in 1971 (separately for urban and rural districts) of the proportions of fathers in different occupations by age of child. These details were used in conjunction with all leukaemia death certificates for children in England and Wales in the period 1970–72. For urban and rural areas separately, the numbers of fathers of children with leukaemia in the three contact categories were compared by age group with those expected on the basis of the proportions of fathers in the general population in those categories.

### RESULTS

Table 1 presents for the highest 'exposure' areas in each population-mixing study, the numbers of children with leukaemia by age group and category of paternal 'occupational contact'. Also given are expected numbers based on the proportions among controls. Table 2 shows significant trends for the pooled data across the categories from 'average plus indeterminate' through 'high' to 'very high', at ages 0–4 years ($P < 0.001$), 5–14 years ($P < 0.01$) and 0–14 years ($P < 0.001$). The incidence of childhood leukaemia associated with fathers in very high (high-child)-contact occupations (all but one of whom were teachers) is twice as high as in the reference (low, medium and indeterminate) category. Other professional occupations did not show these excesses (data not presented), suggesting that they were not related to social class.

When the four broad subgroups within the 'high contact' category (namely sales, 'service and professional', 'transport and communication' and 'construction industry' workers) were examined separately, the excess was found to be most marked in transport- and construction-linked occupations. Compared with the reference category, the preschool children of fathers employed in transport- or construction-linked occupations demonstrated a more than twofold increase in the number of cases of leukaemia.

The findings for construction industry- and transport-related jobs suggest that such occupations are unusually conducive to effective contact with the relevant infection and therefore might appropriately belong in the 'very high' exposure category. However, this extended 'very high' exposure category, being data derived, requires independent testing.

For a wider view of possible associations with paternal contact levels, Table 4 shows the numbers of deaths from childhood leukaemia in the general population of England and Wales in 1970–72, in urban and rural local authority areas separately, by age group and occupational contact category of the father. No appreciable differences are evident between the numbers observed and those expected, based on the corresponding proportions among the fathers (present on census night) of a random sample of all children in the country in 1971.

### DISCUSSION

There is strong evidence that underlying childhood leukaemia is an infection that adults, on occasion, can also transmit. It is plausible, therefore, to consider whether, in microepidemics of this infection (as evidenced by excesses of leukaemia associated with population-mixing), the children of parents with many community contacts or other intense infective exposure are more often, or more severely, infected than others and therefore more likely to develop leukaemia. The present findings support this hypothesis and, moreover, they represent the first such evidence. After a previous allusion (Kinlen and Stiller, 1993) to the present hypothesis concerning paternal occupation, the question of occupational contacts was examined (Roman et al, 1994) in a small case–control study covering most (51) cases in the excess of childhood leukaemia and non-Hodgkin lymphoma in the West Berkshire and North Hampshire Health Districts. No relation was found but it is relevant that the categories of social contact were defined quite differently from those in this study. Thus, in contrast to the present study, there was no 'very high' contact category, while their largest

© Cancer Research Campaign 1997

**Table 2** Childhood leukaemia in five population-mixing studies combined by age group and paternal occupational contact category: observed (and expected) numbers and adjusted O/E ratios

| | 0–4 Years | | | 5–14 Years | | | 0–14 Years | | |
|---|---|---|---|---|---|---|---|---|---|
| **Contact category** | **Obs** | **(Exp)** | **Adjusted O/E** | **Obs** | **(Exp)** | **Adjusted O/E** | **Obs** | **(Exp)** | **Adjusted O/E** |
| Medium and low[a] | 107 | (132.68) | 1.00 | 73 | (83.02) | 1.00 | 180 | (220.70) | 1.00 |
| High | 73 | (49.32) | 1.84 | 54 | (39.60) | 1.55 | 127 | (88.92) | 1.75 |
| Very high | 8 | (4.98) | 2.00 | 5 | (2.38) | 2.39 | 13 | (7.36) | 2.17 |
| Trend (two-sided) | | $P < 0.001$ | | | $P < 0.01$ | | | $P < 0.001$ | |

[a]Including 'indeterminate' occupations. Obs, observed; Exp, expected.

**Table 3** Subdivisions of the high-contact occupation category in population-mixing studies: observed and expected numbers of childhood leukaemia

| | 0–4 Years | | | 5–14 Years | | | 0–14 Years | | |
|---|---|---|---|---|---|---|---|---|---|
| **Contact category** | **Obs** | **Exp** | **Adjusted O/E** | **Obs** | **Exp** | **Adjusted O/E** | **Obs** | **Exp** | **Adjusted O/E** |
| Reference | 107 | (132.68) | 1.00 | 73 | 83.02 | 1.00 | 180 | (220.70) | 1.00 |
| High | 73 | (49.32) | 1.84 | 54 | (39.60) | 1.55 | 127 | (88.92) | 1.75 |
| Sales workers | 8 | (14.06) | 0.71 | 8 | (17.15) | 0.53 | 16 | (31.21) | 0.63 |
| Transport | 23 | (8.04) | 3.55*** | 18 | (4.99) | 4.10*** | 41 | (13.03) | 3.86*** |
| Service and professional | 17 | (18.89) | 1.12 | 17 | (9.23) | 2.10** | 34 | (28.12) | 1.48* |
| Construction industry | 25 | (10.75) | 2.89*** | 11 | (8.16) | 1.53 | 36 | (18.91) | 2.34*** |

Two-sided probabilities: $^{*}P < 0.05$; $^{**}P < 0.01$; $^{***}P < 0.001$.

**Table 4** Childhood leukaemia deaths in England and Wales, 1970–72 by age group and category of paternal occupational contact in urban and rural areas (expected numbers based on proportions in a sample of the general population)

| | 0–4 Years | | | 5–14 Years | | | Total | | |
|---|---|---|---|---|---|---|---|---|---|
| | **Obs** | **(Exp)** | **O/E** | **Obs** | **(Exp)** | **O/E** | **Obs** | **(Exp)** | **O/E** |
| *Urban* | | | | | | | | | |
| Medium and low[a] | 156 | (152.25) | 1.02 | 249 | (265.20) | 0.94 | 405 | (417.45) | 0.97 |
| High | 109 | (109.67) | 0.99 | 199 | (180.58) | 1.10 | 308 | (290.25) | 1.06 |
| Very high | 2 | (5.64) | 0.35 | 9 | (9.95) | 0.90 | 11 | (15.59) | 0.71 |
| Trend (two-sided) | | $P = 0.379$ | | | $P = 0.169$ | | | $P = 0.57$ | |
| *Rural* | | | | | | | | | |
| Medium and low[a] | 45 | (44.68) | 1.01 | 94 | (96.40) | 0.98 | 139 | (141.08) | 0.99 |
| High | 30 | (31.83) | 0.94 | 68 | (64.33) | 1.06 | 98 | (96.16) | 1.02 |
| Very high | 4 | (2.27) | 1.76 | 4 | (4.80) | 0.83 | 8 | (7.07) | 1.13 |
| Trend (two-sided) | | $P = 0.757$ | | | $P = 0.794$ | | | $P = 0.696$ | |

[a]Including 'indeterminate' occupations.

category consisted of low-contact occupations, which, here, formed the smallest. Indeed, the earlier study included in the low-contact group certain jobs connected with transport and the construction industry, which were here assigned to the high contact category. Similarly, the 'medium' contact category in that investigation included certain sales workers who were regarded in the present study as having a high level of contacts. Several other studies have examined parental occupation in relation to leukaemia or to all malignancies in childhood but not in relation to contact levels or to any aspect of infection (Hemminki et al, 1981; Sanders et al, 1981; Van Steensel-Moll et al, 1985; Arundel and Kinnier-Wilson, 1986; Buckley et al, 1989; Ross et al, 1994).

The finding of a high risk associated with construction and transport needs further comment. It is usual on large rural projects

© Cancer Research Campaign 1997

for contractors to subcontract much work to local construction firms, although, inevitably, many other workers have to be specially brought in, often from distant places, but such non-local workers seldom bring their families with them. Similarly, the needs of the construction site bring transport workers temporarily into the local area to service the site, but not accompanied by their families. In contrast, it was appropriate to remove from both case and control groups of the military camp study, the leukaemic children of servicemen, as the proportion of servicemen had defined the grouping of areas for study.

### Large infective dose and/or repeated infection?

The excesses of childhood leukaemia identified by studies of population-mixing have mainly occurred in rural areas. The rural setting suggests that the higher than average proportion of susceptible adults that typifies such areas of low population density is important for the observed effects. It could be argued that if an epidemic of the underlying infection in a rural area was made possible by a relatively high prevalence of susceptible individuals, the occupations highlighted in this study would place some fathers in a position at which a critically high exposure was experienced more often than in urban areas. However, the available data are also compatible with the leukaemogenic exposures being intense, repeated or both. It is relevant that construction workers have also been implicated specifically in previous studies of population mixing and childhood leukaemia. Certain aspects of their work may be particularly conducive to heavy infective exposures. Many live an itinerant life far from home, spending much time, both at leisure and work, in crowded conditions in which hygiene is not a priority. In this connection, it may be relevant that heavy viral exposure is important in feline leukaemia. However, the evidence from population-mixing studies overall is also compatible with repeated exposures being important. Thus, re-infection of immune individuals consequent on repeat exposures occurs with cytomegalovirus infection; indeed, in this case, reinfection involving pregnant women can result in congenital infection, even when the maternal infection is asymptomatic. During mini-epidemics of the infection underlying childhood leukaemia caused by rural population-mixing, it can be assumed that either heavy exposure or repeated infection (or both) would be increased; available evidence does not allow a distinction to be drawn.

### Only in areas of population-mixing?

The findings of the present study need independent confirmation, and this applies particularly to the (negative) findings in the general population, outside areas of unusual population-mixing, as only a crude examination was possible in the present study using sample data from the 1971 census. Thus, no account could be taken of the appreciable proportion of fathers who were absent from home at the census. The evidence of occupation-linked transmission found in this study is derived from rural areas affected by unusual population-mixing and in which excesses of childhood leukaemia have previously been found. In contrast, urban areas with their greater prevalence of immune individuals (particularly adults), consequent on more widespread and earlier exposure, would greatly limit the scope for a severe outbreak of the infection underlying childhood leukaemia; but they would not necessarily prevent, for example, the very high contact category of occupations from showing differences from other occupations.

### Occupation and infection in adults

The present findings provide further confirmation of the infection hypothesis of childhood leukaemia and in particular of the role of adults in transmitting the underlying infection. The magnitude of the excesses and of the trends across the contact categories weigh against chance as the explanation. It is noteworthy that the occupational groups that were associated with the greatest risks of childhood leukaemia have been previously linked with infections. Indeed, recent publications of the Office of Population Censuses and Surveys (OPCS, 1985; OPCS, 1986) have singled out teachers for their unusually frequent exposure to infections that are prevalent among children and students, and an occupational risk of hepatitis among teachers is now recognized under the Industrial Injuries Act. In a different way, lorry drivers, workers in other transport-related occupations and construction workers often have contacts outside their home area and are therefore potentially exposed to different infective agents or, at least, to different strains. It may be relevant that, with the presumption that this is related to frequent absences from home, lorry-driving and construction work are among the occupations that are regularly associated with a high incidence of cervical cancer among the wives of men so employed (OPCS, 1985; OPCS, 1986). The evidence that the increased incidence of cervix cancer is related to an increased exposure by these men to sexually transmitted human papilloma viruses does not imply that such a route of transmission or that these agents are relevant to childhood leukaemia. It does imply, however, that men in these occupations are more likely to have extensive contacts outside their community and that these may allow transmission of other infective agents. Thus, there is nothing essentially implausible about the possibility of unusual infective exposures being related to those occupations.

### Parental occupation and childhood infection

Interest in occupation in relation to infectious diseases has a long history but the relevance of parental occupation to childhood infections is less well known. The present study is by no means the first to report an excess of high contact occupations among the parents of children with an infection-linked disease, as similar observations were made concerning several epidemics of paralytic poliomyelitis. Thus, an excess of bus drivers was noted among the fathers of affected children in a rural outbreak in Essex (Cowan 1950), while in an epidemic in Southend-on-Sea there was a striking excess of schoolteachers among the fathers or household members of patients (Logan 1953). These findings were not confirmed in a later study in London (except for a less marked excess of teacher-fathers) (Benjamin and Logan 1953), although the more strikingly urban setting may be relevant to the negative findings. In a poliomyelitis epidemic in Mauritius McFarlan et al 1946), the incidence was highest among children in the households of men with 'away trades' (lorry and bus drivers, commercial travellers, etc.) than in those of men in 'home trades'. In a study of poliovirus antibody levels in children in relation to social factors, unexpected levels of infection in relation to social class were often associated with fathers in such 'high contact' occupations as teacher, general practitioner, customs official and salesman Backett 1957). These paternal occupational associations with childhood poliomyelitis have additional interest, because this disease is a rare response to a widespread infection that occurs in both children and adults, as childhood leukaemia is postulated to be.

© Cancer Research Campaign 1997

The high prevalence of cytomegalovirus infection among young children in nurseries is now well established as constituting a risk to their care-workers, who may then infect their own children (Stagno et al, 1982, 1984; Preece et al, 1984; Pass et al, 1986, 1987; Alder, 1988; Yow, 1988; Murph et al, 1991). An increased risk of cytomegalovirus infection has also been found among the children of fathers with occupations, or other activities, that involve much contact with children (Stagno et al, 1984). Furthermore, the frequent association of congenital infection with often asymptomatic) maternal cytomegalovirus infection (Stagno et al, 1982; Fowler et al, 1992) suggests an analogy with childhood leukaemia, a disease in which in utero exposures have been considered to be relevant.

### Conclusion

This study finds that, in areas where increases of childhood leukaemia are associated with population mixing, children are at higher risk of childhood leukaemia if their parents are in occupations involving contact with many individuals. No evidence was found that these occupations were associated with excess risk outside areas of high population mixing, although this was based on a limited and indirect examination. The results of this study represent the first individual-based support in childhood leukaemia for infection promoted by population-mixing, as well as further support for the role of adults in transmission of the underlying infection. The findings need confirmation in other examples of population-mixing as do the negative findings outside such special situations. Future studies should compare the totality of contacts of cases and controls (particularly in rural areas), and also test the validity of regarding transport- and construction-linked occupations as being at particular risk, perhaps warranting inclusion in the 'very high' contact category.

### ACKNOWLEDGEMENTS

I am grateful to Dr J Fox, Dr P Goldblatt, Dr M Greenberg, Professor AJ Swerdlow, Professor N Wald and Ms Helen Wilkes for their help in classifying occupations in terms of the estimated levels of contact with different people at work, and to Charles Stiller for providing some of the occupational details.

### REFERENCES

Adler SP (1988) Molecular epidemiology of cytomegalovirus: viral transmission among children attending a day care center, their parents, and caretakers. *J Pediatr* **112**: 366–372

Alexander FE, Chan LC, Lam TH, Yuen P, Leung NK, Ha SY, Yuen HL, Li CK, Li CK, Lau YL and Greaves MF (1997) Clustering of childhood leukaemia in Hong Kong: association with the childhood peak and common acute lymphoblastic leukaemia and with population mixing. *Br J Cancer* **75**: 457–463

Arundel SE and Kinnier-Wilson (1986) Parental occupations and cancer: a review of the literature. *J Epidemiol Commun Hlth* **40**: 30–36

Rackett EM (1957) Social patterns of antibody to poliovirus. *Lancet* **1**: 778–783

Benjamin B and Logan WPD (1953) Geographical and social variations in the incidence of notified poliomyelitis. *Br J Prev Soc Med* **7**: 131–140

Buckley JD, Robison LL, Swotinsky R, Garabrant DH, LeBeau M, Manchester P, Nesbit ME, Odom L, Peters JM, Woods WG, Denman Hammond G (1989) Occupational exposures of parents of children with acute nonlymphocytic Leukaemia: A Report from the Childrens Cancer Study Group. *Cancer Res* **49**: 4030–4037

Cowan HK (1950) *Report of the Medical Officer of Health for Essex for the year 1949*. pp. 66–73

Fowler KB, Stagno S, Pass RF, Britt WJ, Boll TJ and Alford CA (1992) The outcome of congenital cytomegalovirus infection in relation to maternal antibody status. *N Engl J Med* **326**: 663–667.25

General Register Office (1960). *Classification of Occupations*, 1960. HMSO: London.

Hemminki I, Saloniemi I, Salonen T, Partanen T and Vainio H (1981) Childhood cancer and parental occupation in Finland. *J Epidemiol and Commun Hlth* **35**: 11–15

Kinlen LJ (1988) Evidence for an infective cause of childhood leukaemia: comparison of a Scottish new town with nuclear reprocessing sites in Britain. *Lancet* **2**: 1323–1327

Kinlen LJ, Clarke K, Hudson C (1990) Evidence from population mixing in British New Towns 1946–85 of an infective basis for childhood leukaemia. *Lancet* **336**: 577–582

Kinlen LJ and Hudson C (1991) Childhood leukaemia and poliomyelitis in relation to military encampments in England and Wales in the period of national military service, 1950–63. *BMJ* **303**: 1357–1362

Kinlen LJ and Hudson CM and Stiller CA (1991) Contacts between adults as evidence for an infective origin of childhood leukaemia: an explanation for the excess near nuclear establishments in West Berkshire? *Br J Cancer* **64**: 549–554

Kinlen LJ, O'Brien F, Clarke K, Balkwill A and Matthews F (1993*a*) Rural population mixing and childhood leukaemia: effects of the North Sea oil industry in Scotland, including the area near Dounreay nuclear site. *BMJ* **306**: 743–748

Kinlen LJ and John SM (1994) Wartime evacuation of children and mortality from childhood leukaemia in England and Wales in 1945–49. *BMJ* **309**: 1197–1202

Kinlen LJ, Dickson M and Stiller CA (1995) Childhood leukaemia and non-Hodgkin's lymphoma near large rural construction sites, with a comparison with Sellafield nuclear site. *BMJ* **310**: 763–768

Kinlen LJ, Clarke K and Balkwill A (1993*b*) Paternal preconceptional radiation exposure in the nuclear industry and leukaemia and non-Hodgkin's lymphoma in young people in Scotland. *BMJ* **306**: 1153–1158

Kinlen LJ and Stiller C (1993) Rural population mixing and excess of childhood leukaemia. *BMJ* **306**: 930

Langford I (1991) Childhood leukaemia mortality and population change in England and Wales 1969–73. *Soc Sci Med* **33**: 435–440

Logan JS (1953) Poliomyelitis in Southend-on-Sea in 1952. *Proc R Soc Med* **46**: 37–41

McFarlan AM, Dick GWA and Seddon HJ (1946) The epidemiology of the 1945 outbreak of poliomyelitis in Mauritius. *Quart J Med* (New Series) **15**: 183–208

Murph JR, Baron JC, Kice Brown C, Ebelhack CL and Bale Jr JF (1991) The occupational risk of cytomegalovirus infection among day-care providers. *JAMA* **265**: 603–608

Office of Population Censuses and Surveys (1986) *Occupational Mortality: The Registrar General's decennial supplement for Great Britain, 1979–80, 1982–83*. Series DS no. 6. HMSO: London

Office of Population Censuses and Surveys (1995) *Occupational Health: The Registrar General's decennial supplement for England and Wales*. Series DS no. 10. HMSO: London

Pass RF, Hutto C, Ricks R and Cloud G (1986) Increased rate of cytomegalovirus infection among parents of children attending day-care centers. *N Engl J Med* **314**: 1414–1418

Pass RF, Little EA, Stagno S, Britt WJ and Alford CA (1987) Young children as a probable source of maternal and congenital cytomegalovirus infection. *N Engl J Med* **316**: 1366–1370

Petridou E, Revinthi K, Alexander F, Haidas S, Koliouskas D, Kosmidis H, Piperopoulou F, Tzortzatou F and Trichopoulos D (1996) Space-time clustering of childhood leukaemia in Greece: evidence supporting a viral etiology. *Br J Cancer* **73**: 1278–1283

Preece PM, Pearl KN and Peckham CS (1984) Congenital cytomegalovirus infection. *Arch Dis Childhood* **59**: 1120–1126

Roman E, Watson A, Bull D and Baker K (1994) Leukaemia risk and social contact in children aged 0–4 years in southern England. *J Epidemiol Commun Hlth* **48**: 601–605

Ross AR, Davies SM, Potter JD and Robison LL (1994) Epidemiology of childhood leukaemia, with a focus on infants. *Epidemiol Rev* **16**: 243–272

Sanders BM, White GC and Draper GJ (1981) Occupations of fathers of children dying from neoplasms. *J Epidemiol Commun Hlth* **35**: 245–250

Stagno S, Pass RF, Dworsky ME, Henderson RE, Moore EG, Walton PD and Alford CE (1982) Congenital cytomegalovirus infection. The relative importance of primary and recurrent maternal infection. *N Engl J Med* **306**: 945–949

Stagno S, Cloud G, Pass RF, Britt WJ and Alford CA (1984) Factors associated with primary cytomegalovirus infection during pregnancy. *J Med Virol* **13**: 347–353

 © Cancer Research Campaign 1997

Stiller CA and Boyle PJ (1996) Effects of population mixing and socioeconomic status in England and Wales 1979–85, on lymphoblastic leukaemia in children. *BMJ* **313**: 1297–1300

Van Steensel-Moll HA, Valkenburg HA and Van Zanen GE (1985) Childhood leukaemia and parental occupation: a register-based case-control study. *Am J Epidemiol* **121**: 216–224

Yow MD, Williamson DW, Leeds LJ, Thompson P, Woodward RM, Walmus BF, Lester JW, Six HR and Griffiths PD (1988) Epidemiologic characteristics of cytomegalovirus infection in mothers and their infants. *Am J Obstet Gynecol* **158**: 1189–1195

### APPENDIX

### Occupational Contact Categories in terms of 1960 Occupation Codes

*Low:* (Agricultural etc.): 000–007.

*High:* (Sales Workers etc.): 121, 230–235, 237, 239, 276; 'Service and professional' workers: 051, 091, 110, 236, 250–255, 256, 259, 260, 263, 265, 267, 275, 282–285, 294, 298–310, 320, 321; 'Transport and communication' workers: 190–198, 203–206, 208, 209, 266; 'Construction industry': 053, 056, 061, 070, 080, 150, 152–154, 171, 172, 187, 273, 288.

*Very high:* 286, 287.

*Medium* and *Indeterminate:* Allcodes.
Note: Occupations themselves, instead of merely their codes, permit a more sensitive definition of the *Very high* category – see Methods, paragraph 3.

© Cancer Research Campaign 1997